# Genetic variations underlying Gilbert syndrome and HBV infection outcomes: a cross-sectional study

**DOI:** 10.3389/fgene.2023.1265268

**Published:** 2023-11-06

**Authors:** Bilian Yao, Qi Xu, Xinxin Zhang, Yue Han

**Affiliations:** ^1^ Department of General Practice, Ruijin Hospital, Shanghai Jiao Tong University School of Medicine, Shanghai, China; ^2^ Department of Infectious Diseases, Research Laboratory of Clinical Virology, Ruijin Hospital, Shanghai Jiao Tong University School of Medicine, Shanghai, China; ^3^ Sino-French Research Centre for Life Sciences and Genomics, Ruijin Hospital, Shanghai Jiao Tong University School of Medicine, Shanghai, China; ^4^ Clinical Research Center, Ruijin Hospital, Shanghai Jiao Tong University School of Medicine, Shanghai, China

**Keywords:** bilirubin, genotype, Gilbert syndrome, hepatitis B virus, prognosis, protective, *UDP glucuronosyltransferase family 1 member A1*

## Abstract

**Background:** Constant cellular damage causes a poor prognosis of hepatitis B virus (HBV) infection. Accumulating evidence indicates the cytoprotective properties of bilirubin. Here, we investigated the association of *UDP glucuronosyltransferase family 1 member A1* (*UGT1A1*), the genetic cause of Gilbert syndrome (GS), a common condition of mild unconjugated bilirubinemia, with HBV infection outcomes.

**Methods:** Patients (n = 2,792) with unconjugated hyperbilirubinemia were screened for HBV infection and host *UGT1A1* variations in Ruijin Hospital from January 2015 to May 2023, and those with confirmed HBV exposure were included. The promoter/exons/adjacent intronic regions of *UGT1A1* were sequenced. HBV infection outcomes were compared between hosts with wild-type and variant-type *UGT1A1*. The effect magnitudes of *UGT1A1* variations were evaluated using three classification approaches.

**Results:** In total, 175 patients with confirmed HBV exposure were recruited for final analysis. Age, gender, level of HBV serological markers, and antiviral treatment were comparable between *UGT1A1* wild-type and disease-causing variation groups. Five known disease-causing mutations (UGT1A1*28, UGT1A1*6, UGT1A1*27, UGT1A1*63, and UGT1A1*7) were detected. The incidence of cirrhosis or hepatocellular carcinoma (LC/HCC) was significantly lower in *UGT1A1* variant hosts than in *UGT1A1* wild-type hosts (13.14% vs*.* 78.95%, *p < 0.0001*). The rarer the *UGT1A1* variation a patient possessed, the higher the age at which LC/HCC was diagnosed (R = 0.34, *p* < 0.05). In contrast, patients without cirrhosis achieving HBsAg clearance were identified only in the *UGT1A1* variant group (12.32% vs. 0%).

**Conclusion:** The findings of this study provide insights into the association between preexisting genetically mild bilirubin elevation and viral infection outcome. We showed that the accumulation of *UGT1A1* variants or the rarity of the variation is associated with a better prognosis, and the effect magnitude correlates with *UGT1A1* deficiency. This study demonstrates the therapeutic potential of host *UGT1A1* variations underlying GS against HBV infection outcomes.

## 1 Introduction

Bilirubin is the “waste” product of heme catabolism; it is a cause of icterus and can be neurotoxic at high levels. However, at a mildly elevated level, it may orchestrate various biological processes (Vitek, 2020). Bilirubin, unconjugated or conjugated, possesses potent cytoprotective properties (Nakagami et al., 1993). Several studies have explored the benefits of bilirubin since the discovery of its antioxidant properties in the 1950s (Bernhard et al., 1954; Vitek and Schwertner, 2007). Stocker et al. (1987) demonstrated the physiological importance of bilirubin as an antioxidant. Jangi et al. (2013) explained the molecular basis of unconjugated bilirubin in immunomodulation. Furthermore, the benefits of genetically increased levels of unconjugated serum bilirubin against various diseases have also been documented (McCarty, 2007; Schwertner and Vitek, 2008; Horsfall et al., 2014; Wang et al., 2020; Vitek and Tiribelli, 2021; 2023). Vitek and Tiribelli (2021) elaborated on the benefits of mild chronic unconjugated hyperbilirubinemia (Gilbert syndrome, GS) on various comorbidities.

*UDP glucuronosyltransferase family 1 member A1* (*UGT1A1*, OMIM entry number * 191740), on chromosome 2q37, encodes the UGT1A1 enzyme, which plays a key role in bilirubin conjugation; the levels of UGT1A1 are reduced in individuals with GS. Studies have reported the disproportional ethnic distribution of the variants of *UGT1A1*, suggesting local selection pressure. For example, UGT1A1*28 is a polymorphism of the 5´ of the *UGT1A1* gene promoter. The nature of this polymorphism is an insertion of TA pairs. As a known disease-causing variation, homozygotic UGT1A1*28, decreases bilirubin glucuronidation activity, thereby increasing the level of unconjugated bilirubin. The frequency of this disease-causing polymorphism is high in African (Horsfall et al., 2011) and Caucasian (Barbarino et al., 2014) descent but low in Asian and Pacific Island descent (Premawardhena et al., 2003). This phenomenon implies local selection pressure.

Exploring the benefits of mild bilirubinemia as an effective therapeutic strategy to prevent several oxidative stress-induced and inflammatory diseases (Vitek and Tiribelli, 2020) has gained increasing research interest (Vitek et al., 2019). Moreover, the anti-fibrotic effects of hyperbilirubinemia have been demonstrated in a rat model (Wang et al., 2002). The anti-viral properties of biliverdin, an oxidized bilirubin derivative, were documented in the early 1990s (Mori et al., 1991; Nakagami et al., 1992). Additionally, the possible association between biliverdin reductase A expression in peripheral blood leukocytes and treatment response in patients with HCV infection has also been reported (Subhanova et al., 2013). Nevertheless, the role of excessive bilirubin in hepatitis B virus (HBV) infection outcomes remains elusive.

The prevalence of HBV infection is higher and exerts a heavier local disease burden on Asian populations than on those from other parts of the world (GBD2019 Collaborators, 2022). HBV can cause persistent infection, unquenched intrahepatic inflammation, constant cellular damage, and non-cytopathic or even cytopathic effects (Sugiyama et al., 2009) and lead to end-stage events such as liver cirrhosis (LC) or hepatocellular carcinoma [HCC] (Bertoletti and Ferrari, 2016; Iannacone and Guidotti, 2022). Given the multiple benefits of unconjugated bilirubin on cellular protection and immunomodulation, we hypothesized that the prognosis of HBV infection in patients with GS would be better than that in those without GS. To test this hypothesis, we aimed to evaluate the outcome differences associated with variations in *UGT1A1* and explore the association between the mildly elevated unconjugated bilirubin level and the health of an HBV-infected liver.

## 2 Materials and methods

### 2.1 Study population

**FIGURE 1:**
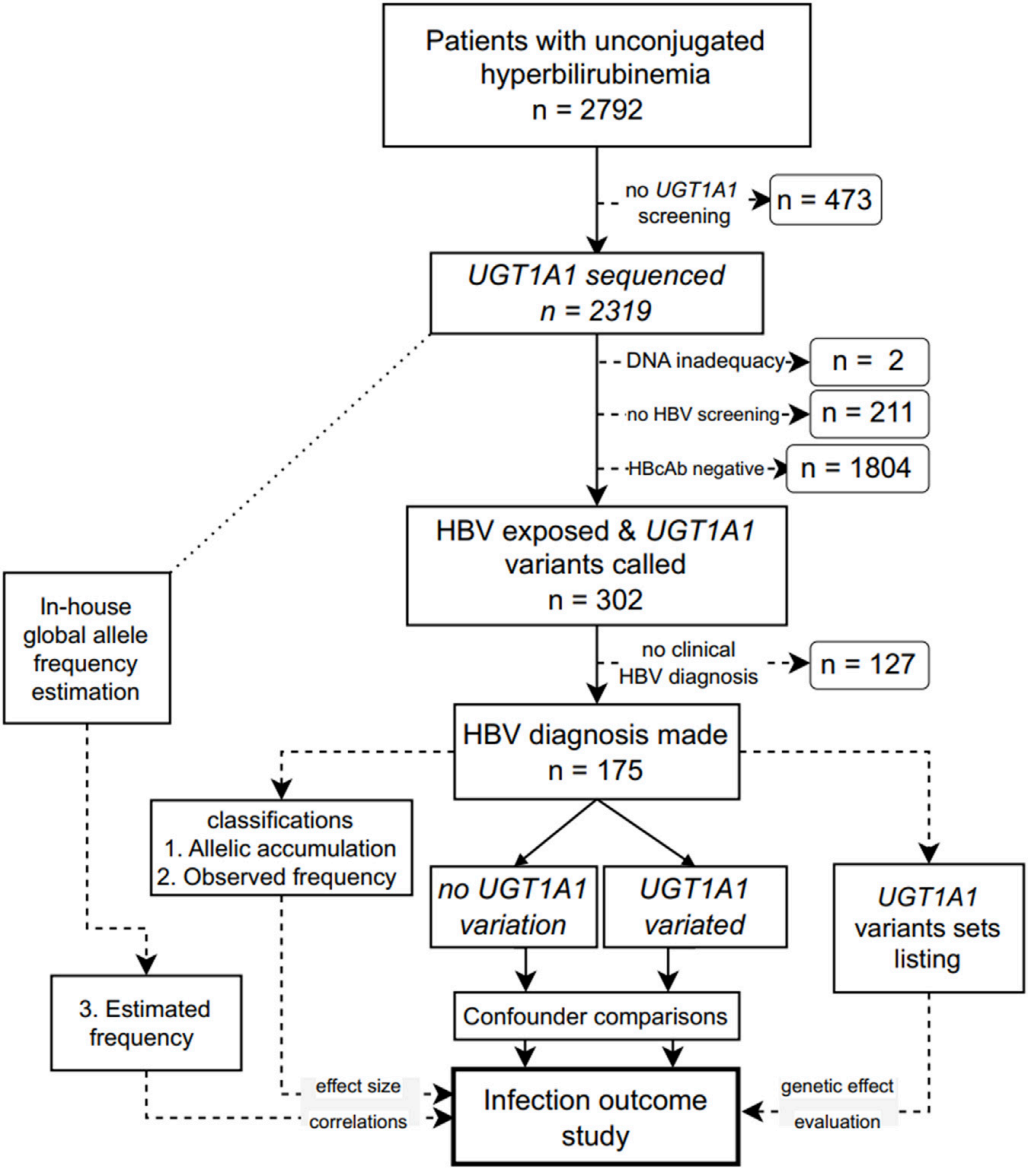
Flowchart of the study design. *UGT1A1*: UDP glucuronosyltransferase family 1 member A1; HBV: hepatitis B virus; HBcAb, an antibody against the HBV core protein.

This single-ethnic study included adult patients (18 years or older) who presented elevated blood unconjugated bilirubin levels with unknown etiology in Ruijin Hospital (Shanghai, China) from January 2015 to May 2023. Patients with known causes of hemolytic anemia, alcohol abuse, Wilson’s disease, autoimmune liver diseases, HBV vaccination, drug usage, known family history of liver cancer, or with co-infection, such as human immunodeficiency virus, hepatitis C virus, hepatitis A virus, hepatitis E virus, hepatitis D virus, or syphilis, were excluded. The remaining patients were screened for *UGT1A1*, and those with known HBV infection outcomes were included in the final analysis (Figure 1). The Ethics Committee of Ruijin Hospital approved this study (approval number 201617). The protocol adhered to the principles of the Declaration of Helsinki and Good Clinical Practice.

### 2.2 Confirmation of HBV exposure and virological testing

Serum HBV markers were determined to confirm HBV exposure in the patients. HBV exposure was defined as having a positive antibody to the HBV core protein (anti-HBc). This antibody, along with an HBV surface antigen (HBsAg), antibody to the HBV surface antigen, hepatitis B e antigen (HBeAg), and its antibody were measured by chemiluminescence using an ARCHITECT i2000SR immunoassay analyzer (Abbott Diagnostics, Abbott Park, IL). HBV DNA levels were determined by real-time PCR using a Cobas Amplicor HBV Monitor Test (Roche Diagnostics, Rotkreuz, Switzerland), with a lower limit of 60 IU/mL, according to the manufacturer’s instructions.

### 2.3 Assessment of HBV infection outcomes

HBV infection outcomes were first classified according to the European Clinical Practice Guidelines (EASL, 2017; EASL, 2018). The five serological phases of the HBV chronic status (phase 1–5), namely, HBeAg-positive chronic infection, HBeAg-positive chronic hepatitis B, HBeAg-negative chronic infection, HBeAg-negative chronic hepatitis B, and the HBsAg-negative phase (with or without cirrhosis), were evaluated. As the development of LC or HCC is a major concern in patients with HBV infection, we further classified the outcome according to the presence or absence of the two events. LC was diagnosed based on either liver biopsy or ultrasound/MRI/CT, with or without supporting evidence derived from non-invasive biochemical markers and transient elastography. HCC was diagnosed by following the criteria outlined in the 2019 guidelines (Xie et al., 2020).

### 2.4 Evaluation of the potential source of bias

Considered as the main confounders, age, gender, HBeAg positivity, and antiviral treatments were subjected to comparison before analyzing the outcome differences between *UGT1A1* wild- and variant-type groups.

### 2.5 Mendelian randomization according to the *UGT1A1* mutational status

Genotyping of the *UGT1A1* sequence was performed according to our previously described protocols (Gu et al., 2022). In brief, all exon, promoter, and adjacent intronic regions of *UGT1A1* were sequenced on a 3500 Dx Genetic Analyzer (Applied Biosystems, Foster City, CA). We retrieved the reference sequence NC_000002.11 (version: GI:224589811) from the RefSeq: NCBI Reference Sequence Database and compared it with the target sequences. CodonCode Aligner (Version 10.0.2) was used to assemble contigs, align to reference, and call variants. We double-confirmed all called variations and their trace signals. Those with known GS-associated genetic variation formed the *UGT1A1* variant group, whereas those without GS-associated genetic variation construed the *UGT1A1* wild-type group.

### 2.6 *UGT1A1* effect magnitude classification

Genetic effect size is positively correlated with the number of deficiency-related mutations and negatively correlated with allelic frequency (Manolio et al., 2009). We first exhaustively listed all the detected variation combinations. However, this approach has its limitations, especially when an equal occurrence rate happens. Hence, we evaluated the *UGT1A1* effect magnitude on infection outcomes based on the following three criteria: (1) variation accumulation, (2) observed and (3) estimated variation combination occurrence.

#### 2.6.1 Number of disease-causing variations: variation accumulation classification

As the pedigree information was lacking, the origin of the variations could not be determined, and we could not count the paternal and maternal deficiency-related variations. Therefore, we classified patients according to their disease-causing locus homozygosity, in addition to the total number of variations compared with the wild-type, into seven groups: (0) no variation (being *UGT1A1* wild-type); (1) one heterozygous variation; (2) two heterozygous variations; (3) three heterozygous variations; (4) one homozygous variated locus; (5) one homozygous locus plus one heterozygous locus; and (6) both loci in a homozygotic state.

#### 2.6.2 Observed variation combination occurrence

Gene effect magnitude negatively correlates with the allelic frequency of each variation. Allele frequency describes how often an allele (a gene variant) appears in a population. We evaluated the effects of allelic frequency on HBV infection outcomes. Although the number of common allelic variations detected was limited to five (Supplementary Table S1), the combination of these five allelic changes resulted in a broader spectrum of possible haplotype frequencies. Therefore, we sorted all observed combinations in descending order according to their occurrences in the studied population (Supplementary Table S2). Subsequently, they were classified into five groups, based on the occurrence from the most common to the rarest, as follows: class 0: *UGT1A1* wild-type; class 1: all combinations that exclusively included the most common allelic changes; class 2: variation combinations that occurred more than five times in the study population; class 3: variation combinations that occurred 2–4 times; and class 4: variation combinations that occurred only once (Supplementary Table S2).

#### 2.6.3 Variation combination occurrence estimated based on in-house minor allele frequency

Since allele occurrence can vary and no Chinese Han allele frequency data on *UGT1A1* are readily available, we used the initial larger *UGT1A1* genotyping in-house data pool. We calculated the in-house minor allele frequency (MAF) based on this dataset, regardless of the HBV infection status as described previously (Conxi Lázaro and Amanda Spurdle, 2021) using the following equation:

$$MAF=\frac{Alleles\;positive\;for\;the\;variant}{Total\;alleles\;screened}.$$

The publicly available MAF data (retrieved from the Database of Short Genetic Variations from the National Library of Medicine, https://www.ncbi.nlm.nih.gov/snp) and in-house estimated MAF of the five most observed loci are given in Supplementary Table S1. Then, the variation occurrence was estimated as the product of MAF of each minor allele. Subsequently, *UGT1A1* variation combinations were categorized into five groups (0–4) according to the estimated incidence—group 0: *UGT1A1* wild type; group 1: >10%; group 2: 1%–10%; group 3: <1%; and group 4: <0.1% (Supplementary Table S2).

### 2.7 Statistical analysis

Preliminary bootstrapped calculations showed that the effect magnitude was >0.5; we chose a modest value of 0.25. The sample size was estimated with a significance level of 0.05 and a power of 0.8 using the *pwr.chisq.test* function of the *pwr* R package—the optimal size was determined to be 155. Continuous variables were subjected to normality checks, and the downstream analyses using parametric (*Student’s t*) or nonparametric (*Wilcoxon*) tests were performed depending on the normality status. Pearson’s *chi-squared* test with a *Yate’s* continuity correction was used to test the goodness-of-fit of whether a categorical variable followed a hypothesized distribution. The *Kruskal–Wallis* test was used to differentiate values among multiple groups. The regression analyses were performed using linear models. All tests were two-sided, with a significance level of <0.05. All statistical analyses were performed using *RStudio* (version 2023.03.0 + 386″ Cherry Blossom” Release, Massachusetts, United States of America).

## 3 Results

### 3.1 Comparable demographics between *UGT1A1* wild-type and variant groups

After excluding patients following the exclusion criteria (Figure 1), 175 patients were included in the final analysis. Detailed characteristics of the patients grouped into the *UGT1A1* wild-type and variant groups based on the sequencing and genotyping data of *UGT1A1* are listed in Table 1. Age and gender were comparable between the *UGT1A1* wild-type and variant groups. The number of female patients was low in both groups. Viral load, HBeAg positivity, and antiviral treatment were similar between the *UGT1A1* wild-type and variant groups. Details about serum bilirubin levels are given in Supplementary Tables S3–S6.

**TABLE 1:** Demographic comparison of *UGT1A1* groups.

			*UGT1A1* wild type n = 38							*UGT1A1* variant n = 137				*p*-value	
Age (years, mean)			45.29							41.42				0.1114	
Gender (female/male)			10/28							31/106				0.7960	
HBeAg (positive/negative)			8/30							27/110				1.0000	
Viral load (detectable/negative)			18/17							45/92				0.0797	
Antivirals (treated/naïve)			18/20							67/70				1.0000	

*UGT1A1* variation accumulation classification															
			wt^a^	Class 1			Class 2	Class 3		Class 4		Class 5	Class 6		
Age (years, mean)			45.29	42.07			41.93	45.27		39.07		40	40	*0.5010*	
Gender (female/male)			10/28	7/34			11/30	3/8		8/22		2/10	0/2	*0.8631*	

Observed variation combination occurrence classification															
			wt^a^			Class 1^b^			Class 2		Class 3		Class 4		
Age (years, mean)			45.29			41.22			43.62		46.13		33.90	0.04149	
Gender (female/male)			10/28			19/71			9/20		1/7		2/8	0.7396	

Estimated variation combination occurrence classification															
			wt^a^			Class 1			Class 2		Class 3		Class 4		
Age (years, mean)			45.29			41.69			41.08		43.09		36.33	0.2712	
Gender (female/male)			10/28			7/29			12/47		11/22		1/8	0.4972	

^a^ wt, wild type.

^b^ Classes 1–4 represent a descending order of occurrence.

### 3.2 *UGT1A1* variation combination occurrence correlated with LC or HCC diagnosis age

**FIGURE 2:**
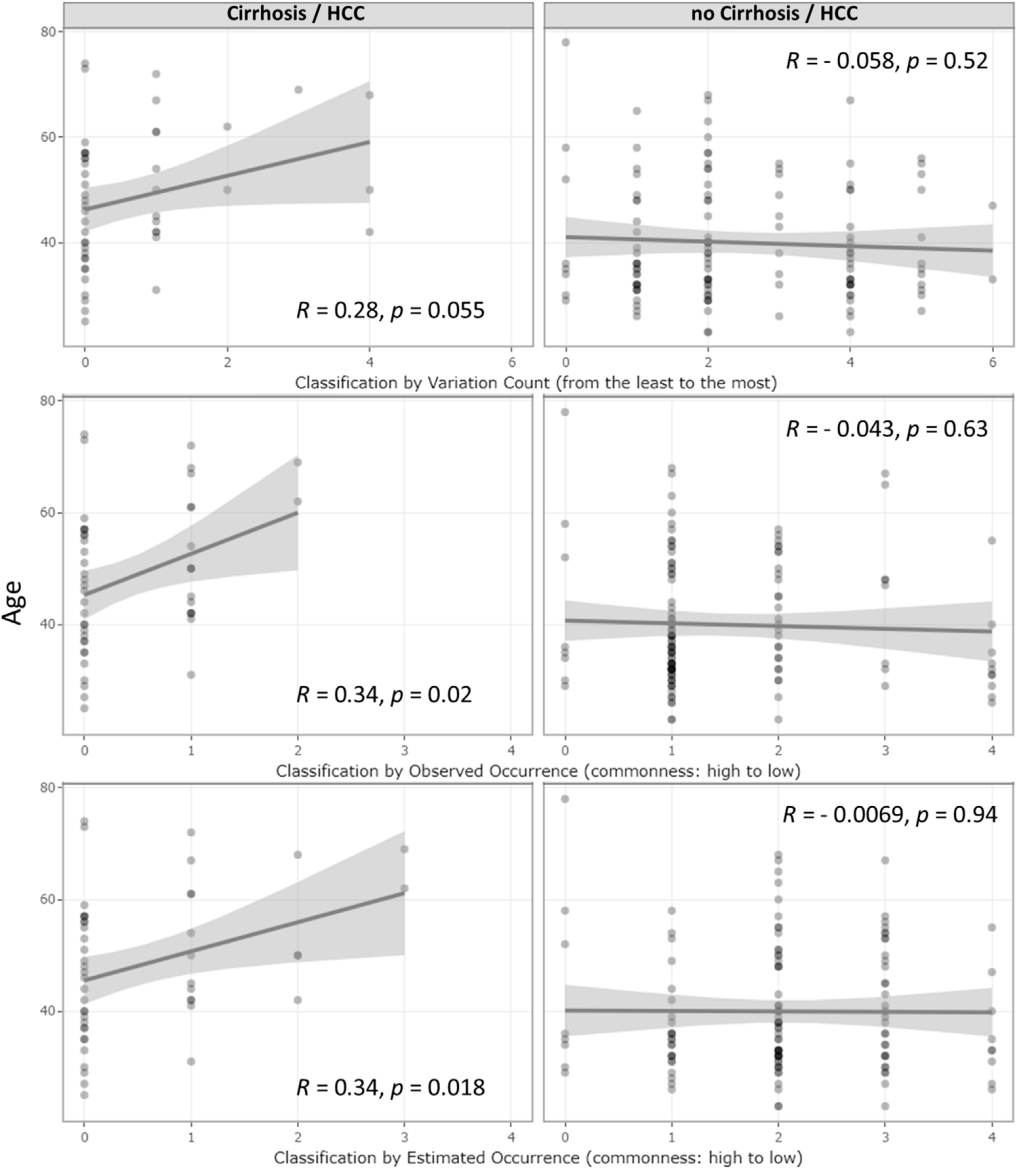
Correlation of age at the diagnosis of HBV infection outcomes and *UGT1A1* genetic effect classifications. Correlations of the underlying genetic deficiency with LC/HCC diagnosis age and their corresponding R- and *p*-values. The three rows represent the three genetic deficiency classifications: variation accumulation, observed variation combination occurrence, and estimated occurrence based on in-house MAF. The left and right column graphs show the result from the LC/HCC subset and non-LC/HCC subset, respectively. The *x*-axis represents the genetic effect magnitude of the genetic deficiency, increasing from left to right.

Even though no age difference was observed between the *UGT1A1* variant and wild-type groups, within the LC/HCC diagnosed subsets, patients with *UGT1A1* wild type developed LC/HCC at a younger age (*p = 0.054*). As shown in Figure 2, correlation tests revealed that accumulation of variations was positively associated with LC/HCC diagnosis age (R = 0.28, *p = 0.055*). Observed variation combination occurrences in descending order were positively associated with diagnosis age (R = 0.34, *p = 0.02*). Furthermore, the estimated occurrence classification also demonstrated a positive correlation (R = 0.34, *p = 0.018*). In contrast, no correlation with the diagnosis age was observed in non-LC/HCC subgroups.

### 3.3 LC or HCC events overpresented in the *UGT1A1* wild-type group (patients without GS)

**FIGURE 3:**
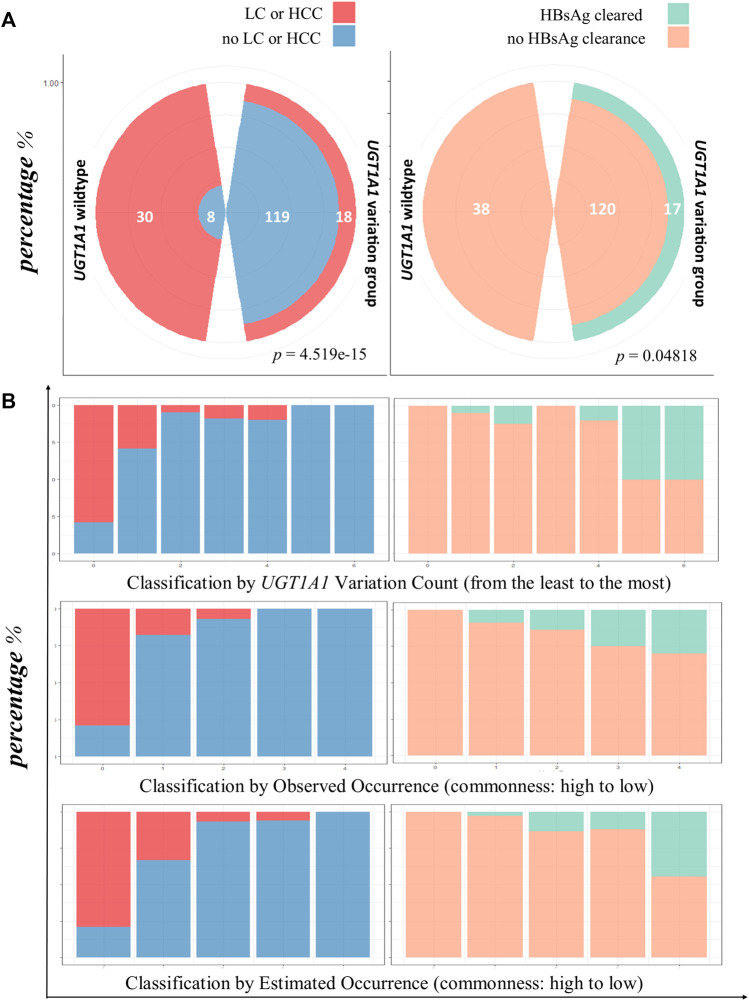
HBsAg clearance and lower LC/HCC associated with *UGT1A1* variation. **(A)** Comparison of infection outcomes between *UGT1A1* wild-type and variant groups. The left polar graph shows the proportion of occurrence or no occurrence of LC/HCC in patients with *UGT1A1* wild type or variant type; the right panel shows clearance or no clearance of HBsAg in patients with the *UGT1A1* wild type or variant type. **(B)** Bar chart view of LC/HCC or HBsAg clearance diagnosis using three genetic effect classifications. The first row shows the results for variation accumulation classification; the *x*-axis represents the increase in variation accumulation from left to right. The left-side graphs (in blue and red) represent LC/HCC diagnosis, and the right-side graphs (orange and green) show HBsAg clearance. The second row shows the observed frequency classification, decreasing from left to right. The last row depicts the diagnosis proportion with the estimated frequency classification, where the frequencies decrease from left to right.

The incidence of LC or HCC varied between the patients with the *UGT1A1* wild type and variant type (Mendelian randomization). The rate of incidence of LC or HCC was significantly higher in the *UGT1A1* wild-type group (*p = 4.519e−15*, Figure 3).

### 3.4 Non-cirrhotic HBsAg clearance is exclusively observed in patients with GS

Next, we assessed the rate of HBsAg clearance in patients without cirrhosis. In the *UGT1A1* wild-type group, no HBsAg clearance occurred. However, 17 patients exhibited HBsAg clearance in the *UGT1A1* variant group *(p = 0.04818*; Figure 3A).

### 3.5 Low occurrence of *UGT1A1* variation combination associated with better outcomes

To evaluate the effect size of *UGT1A1* variations on infection outcomes, we first calculated the percentage of either HBV serological phasing, LC/HCC, or HBsAg clearance in all 23 observed *UGT1A1* variation combinations (including the wild type) and directly sorted them in descending order according to their occurrences, with no classification. As shown in Supplementary Figure S1, LC/HCC gradually disappeared with decreasing *UGT1A1* variation combination occurrence, while cases of HBsAg clearance increased.

### 3.6 Infection outcome protection evidenced by additional variation classifications

Next, we evaluated the effect size of *UGT1A1* variation number and combination occurrence classes. The rate of incidence of LC/HCC was reduced with increased variation accumulation (*p = 1.018e−13*). Similar trends of reduced occurrence of LC/HCC were determined with observed (*p* = *6.529e−14*) and estimated (*p* = *2.083e−15*) variation combination frequency classifications. However, the HBsAg-cleared cases showed contrasting results (Figure 3).

## 4 Discussion

With the discovery of new complex characteristics of bilirubin over recent decades, the therapeutic potential of mildly elevated unconjugated bilirubinemia, especially GS, has become a research focus. A few studies have even proposed not labeling individuals with GS as “patients” (Vitek and Tiribelli, 2021). Nevertheless, the level of bilirubin is affected by nutrition, hormonal, drug, and exercise status, along with several other factors. Moreover, besides its association with liver diseases, bilirubinemia can be a pathological consequence. Therefore, establishing a direct association between bilirubin and liver disease is challenging. On the contrary, genetic or Mendelian randomization (Emdin et al., 2017) can eliminate the environmental, social, or physical confounders as the host gene is predefined and stable. Therefore, instead of using biochemical phenotyping, in this study, we investigated the association between GS and HBV infection outcomes using *UGT1A1* genotyping and explored the therapeutic potential of hyperbilirubinemia against HBV infection for the first time.

The findings of this study confirm that upon HBV exposure, the prognosis in hosts with or without *UGT1A1* variation differs significantly. With subsequent analyses using various grouping approaches, we revealed that the accumulation of variations is associated with improving outcomes. The same was observed with the decreasing occurrences of specific variation combinations, observed or estimated using in-house larger sample MAF calculation. These findings qualitatively and quantitatively support the benefits conferred by “bilirubin disorder.” The deficiency of *UGT1A1* has been speculated to have evolutionary benefits as its polymorphisms are unequally distributed among *Homo sapiens* populations (Wagner et al., 2018). One of the common polymorphisms, a homozygous form called UGT1A1*28, occurs in 10%–25% of individuals of African and Indian subcontinent descent, with a variable frequency in Europe and much lower frequency in Southeast Asia, Melanesia, and the Pacific Islands [ranging from 0% to 5%] (Premawardhena et al., 2003). Moreover, the global frequency of *UGT1A1* variations differs from those reported regionally. However, the underlying causes of these differences need to be further tested.

In terms of potential benefits of bilirubin to the HBV-infected liver, a study demonstrated that mild unconjugated hyperbilirubinemia is associated with a decreased risk of non-alcoholic fatty liver disease (Weaver et al., 2018). Moreover, bilirubin might be a ligand of the liver-specific α1-fetoprotein and has been speculated to be involved in cell growth, differentiation, and regeneration (Vitek and Tiribelli, 2021).

As the first exploratory study, the present study has some limitations. In the *UGT1A1* variation dosage classification, owing to limited pedigree information, allelic phasing was difficult. The haplotype of multiple heterozygous loci can either be heterozygous or compound heterozygous. Hence, their positioning in the scaling was less representative. Therefore, future studies, including parental genetic information, are necessary for a more straightforward classification of haplotypes. Additionally, hepatitis viruses other than HBV were not studied owing to data insufficiency. However, with the few hepatitis A, D, or E virus-infected cases encountered, liver failure coincided with the *UGT1A1* wild-type host; nevertheless, a detailed investigation is essential.

## Data Availability

The original contributions presented in the study are included in the article/Supplementary Material; further inquiries can be directed to the corresponding authors.
