# Supplementary material for: Genetic variations underlying Gilbert syndrome and HBV infection outcomes: a cross-sectional study
Source: Front Genet. 2023 Nov 6;14:1265268. doi: 10.3389/fgene.2023.1265268 (PMC10657892; doi:10.3389/fgene.2023.1265268)
Supplement: Supplementary file 1 [file Table1.DOCX]

Supplementary Material

# Supplementary Figures and Tables

## Supplementary Table 1: Five most common single loci frequencies

| **Nomenclature** | **rs no.** | **Variation** | **dbSNP MAF***  **(population)** | **Inhouse UGT1A1 testing MAF** |
| --- | --- | --- | --- | --- |
| UGT1A1*28 | *rs3064744* | dupTA | 0.089 (Vietnamese) | 0.252416 |
|  |  |  | 0.3253  (1000G**) |  |
| UGT1A1*6 | *rs4148323* | Gly71Arg | A = 0.18017  (Japan) | 0.17881 |
|  |  |  | A = 0.0343 (1000G) |  |
| UGT1A1*27 | *rs35350960* | Pro229Gln | A = 0.00488  (Japan) | 0.033271 |
|  |  |  | A = 0.0028 (1000G) |  |
| UGT1A1*63 | *rs34946978* | Pro364Leu | T = 0.00418  (Japan) | 0.031413 |
|  |  |  | T = 0.0022 (1000G) |  |
| UGT1A1*7 | *rs34993780* | Tyr486Asp | G = 0.00131  (Japan) | 0.015613 |
|  |  |  | G = 0.0008 (1000G) |  |

**dbSNP: Single Nucleotide Polymorphism database*

*MAF: Minor allele frequency*

***1000G: The 1000 Genomes Project*

## Supplementary Table 2: Detected *UGT1A1* gene variation combination counts and classifications

| **Variation type** | **Count** | **Observed frequency class** | **Estimated incidence based on inhouse MAF** | **Estimated class**** |
| --- | --- | --- | --- | --- |
| wildtype | 35 | wildtype | wildtype | wildtype |
| TA7Gly71ArgHet2 | 24 | 1 | 0.045134672 | 2 |
| Gly71ArgHet | 19 | 1 | 0.178810409 | 1 |
| TA7Hom | 18 | 1 | 0.063714017 | 2 |
| TA7Het | 16 | 1 | 0.252416357 | 1 |
| Gly71ArgHom | 10 | 1 | 0.031973162 | 2 |
| TA7Pro229GlnHomHet | 10 | 2 | 0.002119853 | 3 |
| TA7Gly71ArgPro229GlnHet3 | 9 | 2 | 0.001501693 | 3 |
| Gly71ArgPro364LeuHet2 | 5 | 2 | 0.005616907 | 3 |
| TA7Pro364LeuHet2 | 5 | 2 | 0.007929064 | 3 |
| Pro364LeuHet | 4 | 3 | 0.031412639 | 2 |
| TA7Pro229GlnHet2 | 2 | 3 | 0.008398239 | 3 |
| TA7Pro229GlnHom2 | 2 | 3 | 7.05304E-05 | 4 |
| Gly71ArgPro364LeuTyr486AspHet3 | 1 | 4 | 8.76989E-05 | 4 |
| Gly71Argrs4148327Het2 | 1 | 4 | <0.178810409 | 4 |
| Gly71ArgTyr486AspHet2 | 1 | 4 | 0.002791835 | 3 |
| Gly71ArgTyr486AspHetHom | 1 | 4 | 4.359E-05 | 4 |
| TA7Gly71ArgTyr486AspHet3 | 1 | 4 | 0.000704705 | 4 |
| TA7IntronnovelHet2 | 1 | 4 | <0.252416357 | 4 |
| TA7P82SnovelHomHet | 1 | 4 | <0.0637140172811514 | 4 |
| TA7Tyr486AspHet2 | 1 | 4 | 0.003941073 | 3 |
| Tyr486AspHet | 1 | 4 | 0.015613383 | 2 |
| Tyr486AspHom | 1 | 4 | 0.000243778 | 4 |

**Classification criteria from class 1 to 4. Class 1 represents all the combinations that exclusively included the most common two loci; class 2 represents genotypes occurring more than five times; class three represents genotypes appearing two–four times; class 4 represents genotypes occurring only once.*

*** Classification was made according to frequency: (1) > 10%, (2) 1%–10%, (3) < 1%, (4) <1‰.*

**Supplementary Table 3: Pairwise comparisons of total bilirubin levels for each *UGT1A1* genotype compared to those with the wild-type**

|  | **Total bilirubin level (μmol/L)** | | **p-value** (Wilcoxon test) |
| --- | --- | --- | --- |
| **Genotype** | **Median value** | **Interquartile range value** |  |
| Arg367HisHet | 27.5 | 0 | 0.9632479 |
| Gly71ArgAsn400HisHomHet | 21.3 | 0 | 1 |
| Gly71ArgHet | 28 | 33.3 | 0.528356224 |
| Gly71ArgHetVUS | 13.5 | 19.2 | 0.82383041 |
| Gly71ArgHom | 29.3 | 19.3 | 0.159090718 |
| Gly71ArgHomVUS | 29.2 | 9.5 | 0.81005096 |
| Gly71ArgPro364LeTyr486AspuHet3 | 34.3 | 0 | 0.9632479 |
| Gly71ArgPro364LeuHet2 | 27.75 | 19 | 0.7210992 |
| Gly71ArgPro364LeuTyr486AspHet3 | 64.2 | 0 | 0.8238304 |
| Gly71ArgPro387SerHet2VUS | 48.5 | 0 | 0.8879673 |
| Gly71ArgTyr486AspHet2 | 25.9 | 13.375 | 1 |
| Gly71ArgTyr486AspHetHom | 159.1 | 48.025 | 0.15909072 |
| Gly71ArgTyr486AspHom2 | 202.15 | 0.45 | 0.3808619 |
| Gly71ArgTyr486AspHomHet | 28.8 | 0 | 0.9632479 |
| P364LHet | 11.2 | 0 | 0.8320526 |
| Pro229GlnHet | 364.9 | 0 | 0.7148541 |
| Pro229GlnPro364LeuHet2 | 26.15 | 6.75 | 1 |
| Pro364LeuHet | 22.6 | 17.275 | 1 |
| Pro364LeuHetVUS | 54.4 | 0 | 0.8320526 |
| Pro364LeuHom | 19.9 | 0 | 1 |
| Pro364LeuTyr486AspHet2 | 30.95 | 2.55 | 0.9049205 |
| TA7Gly71ArgHet2 | 31.4 | 15.65 | 0.001238546 |
| TA7Gly71ArgHet2VUS | 31.9 | 0 | 0.8948023 |
| TA7Gly71ArgHetHom | 50.9 | 0 | 0.854687 |
| TA7Gly71ArgPro229GlnHet3 | 31.25 | 14.825 | 0.3009998 |
| TA7Gly71ArgPro229GlnTyr486AspHet4 | 35.4 | 0.1 | 0.8238304 |
| TA7Gly71ArgTyr486AspHet3 | 65.55 | 4.65 | 0.7210992 |
| TA7Het | 27.7 | 31.825 | 0.257110123 |
| TA7HetVUS | 28.9 | 37 | 0.9071033 |
| TA7Hom | 38.9 | 27.35 | 1.20E-06 |
| TA7HomVUS | 60.7 | 0 | 0.8238304 |
| TA7Pro229GlnHet2 | 30.4 | 29.025 | 0.3186667 |
| TA7Pro229GlnHom2 | 45.7 | 12.975 | 0.6387675 |
| TA7Pro229GlnHom2VUS | 29.8 | 0 | 0.9632479 |
| TA7Pro229GlnHomHet | 45.05 | 18.925 | 0.001596606 |
| TA7Pro229GlnPro364LeuHet3 | 22.1 | 4.3 | 1 |
| TA7Pro364LeuHet2 | 29.6 | 9.8 | 0.1573445 |
| TA7Pro364LeuHet2VUS | 68.3 | 12.2 | 0.7149115 |
| TA7Pro364LeuTyr486AspHet3 | 75 | 0 | 0.8172589 |
| TA7Tyr486AspHet2 | 56.7 | 101.275 | 0.3298292 |
| TA7Tyr486AspHomHet | 41 | 0 | 0.9558514 |
| TA7Val491MetHomHet | 60.7 | 0 | 0.8238304 |
| Tyr486AspHet | 26.5 | 9.7 | 1 |
| Tyr486AspHetVUS | 54 | 43.25 | 0.9632479 |
| Tyr486AspHom | 56.45 | 15.3 | 0.3009998 |
| VUS | 24.75 | 38.025 | 0.8948023 |
| WT | 22.9 | 32.8 | NA |

**Supplementary Table 4: Comparisons of total bilirubin levels between *UGT1A1* wild-type and variated groups and further comparisons between HBV infection subgroups**

| **UGT1A1** | **HBV** | **Total bilirubin level (μmol/L)** | | **p-value** (Wilcoxon test) | |
| --- | --- | --- | --- | --- | --- |
|  |  | **Median value** | **IQR value** |  |  |
| **Variated** | **All** | 31.3 | 25.4 |  | 6.669e-07 |
|  | HBV | 30.3 | 18.975 | 0.56344075 |  |
|  | No HBV | 31.5 | 26.7 |  |  |
| **Wildtype** | **All** | 22.9 | 32.8 |  |  |
|  | HBV | 43.5 | 131.5 | 0.002990439 |  |
|  | No HBV | 22.15 | 21.925 |  |  |

**Supplementary Table 5: Comparisons of total bilirubin levels between variated and non-variated *UGT1A1* probands diagnosed with liver cirrhosis or hepatocellular carcinoma**

| **UGT1A1** | **Total bilirubin level (μmol/L)** | | **p-value** (Wilcoxon test) |
| --- | --- | --- | --- |
|  | **Median value** | **IQR value** |  |
| **Variated** | 57.85 | 61.675 | *0.7759* |
| **Wildtype** | 50.95 | 68.625 |  |

**Supplementary Table 6: Comparisons of total bilirubin levels in those who cleared the virus versus those without clearance among variated *UGT1A1* probands**

| **UGT1A1** | **Total bilirubin level (μmol/L)** | | **p-value** (Wilcoxon test) |
| --- | --- | --- | --- |
|  | **Median value** | **IQR value** |  |
| **HBsAg cleared** | 29.70 | 19.9 | 0.6737 |
| **No clearance** | 29.85 | 14.7 |  |


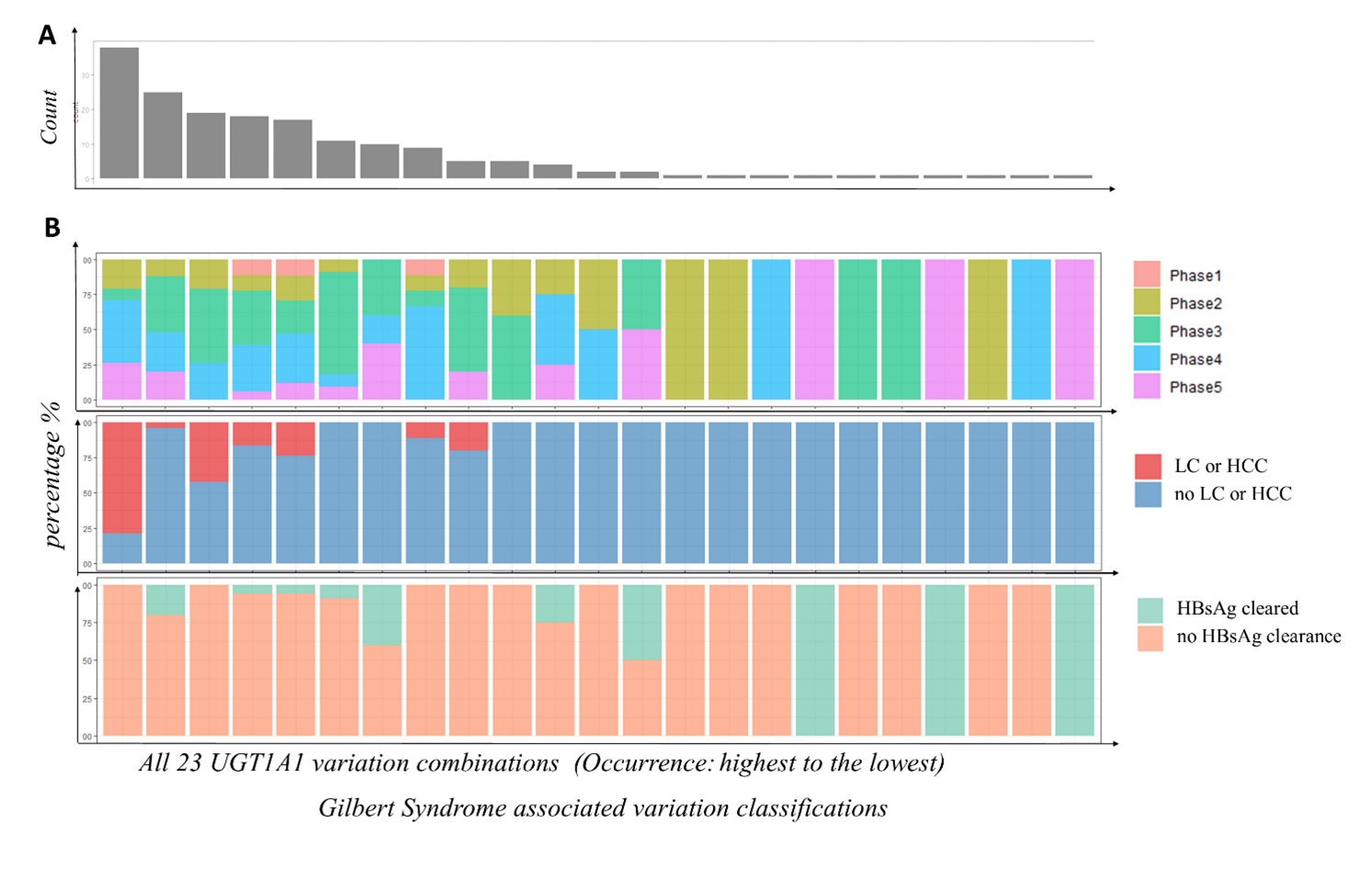


## Supplementary Figure 1: HBsAg clearance and lower incidences of liver cirrhosis/hepatocellular carcinoma are associated with *UGT1A1* variation

**(A)** Occurrences of every genotype combination detected in the study groups listed in a descendant manner from left to right. The leftmost is the *UGT1A1* wildtype patient counts; **(B)** The top graph shows the composition of HBV infection phases of each *UGT1A1* genotype combinations. The second graph demonstrates the proportion of LC/HCC diagnosis. The third graph shows the HBsAg clearance diagnosis. The genotype combination of each bar corresponds to the bar above in panel (A).
